# Small-area estimation for public health surveillance using electronic health record data: reducing the impact of underrepresentation

**DOI:** 10.1186/s12889-022-13809-2

**Published:** 2022-08-09

**Authors:** Tom Chen, Wenjun Li, Bob Zambarano, Michael Klompas

**Affiliations:** 1 Department of Population Medicine, Harvard Medical School and Harvard Pilgrim Health Care Institute, Boston, MA USA; 2 Department of Public Health, University of Massachusetts Lowell, Lowell, MA USA; 3 Commonwealth Informatics, Waltham, MA USA; 4 Department of Medicine, Brigham and Women’s Hospital, Boston, MA USA

**Keywords:** Behavioral risk factor surveillance system, Population surveillance, Asthma, Diabetes mellitus, Hypertension, Obesity, Smoking

## Abstract

**Background:**

Electronic Health Record (EHR) data are increasingly being used to monitor population health on account of their timeliness, granularity, and large sample sizes. While EHR data are often sufficient to estimate disease prevalence and trends for large geographic areas, the same accuracy and precision may not carry over for smaller areas that are sparsely represented by non-random samples.

**Methods:**

We developed small-area estimation models using a combination of EHR data drawn from MDPHnet, an EHR-based public health surveillance network in Massachusetts, the American Community Survey, and state hospitalization data. We estimated municipality-specific prevalence rates of asthma, diabetes, hypertension, obesity, and smoking in each of the 351 municipalities in Massachusetts in 2016. Models were compared against Behavioral Risk Factor Surveillance System (BRFSS) state and small area estimates for 2016.

**Results:**

Integrating progressively more variables into prediction models generally reduced mean absolute error (MAE) relative to municipality-level BRFSS small area estimates: asthma (2.24% MAE crude, 1.02% MAE modeled), diabetes (3.13% MAE crude, 3.48% MAE modeled), hypertension (2.60% MAE crude, 1.48% MAE modeled), obesity (4.92% MAE crude, 4.07% MAE modeled), and smoking (5.33% MAE crude, 2.99% MAE modeled). Correlation between modeled estimates and BRFSS estimates for the 13 municipalities in Massachusetts covered by BRFSS’s 500 Cities ranged from 81.9% (obesity) to 96.7% (diabetes).

**Conclusions:**

Small-area estimation using EHR data is feasible and generates estimates comparable to BRFSS state and small-area estimates. Integrating EHR data with survey data can provide timely and accurate disease monitoring tools for areas with sparse data coverage.

**Supplementary Information:**

The online version contains supplementary material available at 10.1186/s12889-022-13809-2.

## Background

Electronic Health Record (EHR) data are increasingly being used for public health surveillance by local and national public health jurisdictions. Although data from EHRs are primarily used to manage individuals’ health, they have the potential to facilitate population health surveillance. EHRs cover far more people than traditional public health surveys, and once the infrastructure is in place, EHR data can be refreshed and analyzed in real-time without additional recruitment or interviewing costs [1]. In the past few years [2], several jurisdictions have made enormous progress in accessing and integrating electronic health data from various sources, including Chicago’s Health Atlas [3] and New York’s Macroscrope [4]. This work will focus on chronic disease prevalence estimation using data from Massachusetts’s MDPHnet [5].

Despite the advantages of EHR data over health surveys, estimating disease prevalence using EHR data remains subject to question. The first challenge is the same faced by health surveys in that, despite having numerically higher coverage rates than traditional health surveys in catchment areas of the healthcare system, geographic areas outside the catchment areas are usually underrepresented, therefore direct estimates for these areas are not available and/or not reliable. The second challenge is that the data is not randomly sampled and therefore may not represent the target population. For example, the patient population from MDPHnet overrepresents selected minorities and patients living in high poverty neighborhoods and underrepresents patients living in small rural communities. While MDPHnet has been demonstrated to return statewide estimates consistent with estimates from the Centers for Disease Control and Prevention’s (CDC’s) Behavioral Risk Factor Surveillance System (BRFSS) [6], it remains unclear how MDPHnet performs for local municipalities.

In this paper, we explore the use of small-area estimation (SAE) for disease prevalence surveillance. We integrate individual-level clinical and demographic data from the EHR with community-level socioeconomic data from the American Community Survey and community-level hospitalization data from the state health department. SAE methods “borrow” information from other communities by linking key socio-demographic information to allow estimates to be transferable to off-sampled communities. They have been applied in many contexts with traditional survey data, such as obesity [7], tobacco use [8], COPD [9], and periodontitis [10], and in the more recent CDC project Places: Local Data for Better Health [11]. We sought to extend the techniques of SAE to EHR data for five health conditions — asthma, diabetes, hypertension, obesity, and smoking. We evaluated the performance of increasingly-refined models (i.e. the effect of weighting and inclusion of more predictors in the model) with comparisons to BRFSS 500 Cities estimates for Massachusetts cities and towns. Equipped with this predictive model, we identified the municipalities at greatest risk for the aforementioned health conditions to demonstrate its potential utility for operational public health surveillance.

## Methods

SAE methods can be broadly divided into design-based methods (estimates constructed from the sampling design) and model-based methods (estimates relying on a specified model) [12]. However, design-based methods fail for undercovered areas, and for these areas, only a model-based method can be utilized. Multilevel regression and poststratification (MRP) is a model-based SAE approach that was first used to estimate state-level indicators from national polls and has since been extended to track an array of health outcomes from traditional surveys, notably by the CDC’s PLACES project [11]. Additionally, the model-based nature of MRP can adjust the estimation from a nonrepresentative survey as a result of recruiting difficulties [13]. MRPs do not need to be restricted to just survey data — respresentative or not — but can also be applied to EHR data, which can be viewed as a highly nonrepresentative survey. We apply MRP techniques to five health conditions — asthma, diabetes, hypertension, obesity, and smoking — and to evaluate their performance against BRFSS’ 500 Cities estimates for Massachusetts cities and towns. We will first introduce the data sources employed in this study, and then describe the details of our MRP.

### Data sources

MDPHnet: MDPHnet [5] is a distributed health data network in Massachusetts that permits authorized public health officials to submit detailed queries against the EHR data of three large multispecialty group practices that serve a combined patient population of approximately 1.1 million people (about 19% of the state population as of 2020). Each practice group uses open-source software (Electronic Medical Record Support for Public Health) [14] to create, host, and maintain a data repository behind its firewall in accordance with a common data specification. MDPHnet’s current practice partners include Atrius Health, a large multisite, multispecialty ambulatory group serving primarily a well-insured population (approximately 800,000 patients, 29 clinical locations), the Massachusetts League of Community Health Centers (MLCHC), a network of community health centers targeting underserved populations (approximately 500,000 patients, 15 clinical locations), and Cambridge Health Alliance (CHA), a combination safety net–general practice hospital and ambulatory group (approximately 200,000 patients, 15 clinical locations). The overall patient return rate across all 3 practice groups is 82% [15]. Analysis for this study was reviewed and approved by the institutional review board of Harvard Pilgrim Health Care Institute.

We analyzed 5 test conditions — diabetes, asthma, smoking, hypertension, and obesity — using EHR-data derived from MDPHnet (disease definitions are listed in Additional file 1). To facilitate comparisons against BRFSS 500 Cities estimates conducted in 2016, we restricted our analysis to anonymized, individual-level monthly data from MDPHnet from 2016 on individuals aged ≥20 with at least one outpatient visit of any kind in the health care system in the 2 years prior to each index month in 2016. The individual-level characteristics available within MDPHnet are sex, race, and age group. Because MDPHnet has measurements taken each month, we aggregated all 12 months of data for 2016 and included a time trend variable to account for temporal changes. Note that each provider will carry over mostly the same set of patients each month, although some patients may change providers and patient IDs were not provided for this analysis, making it impossible to account for within-person correlations over the months. Therefore, while we utilize all 12 months of data for estimation purposes, we inflated standard errors as if we had 1 month of data to provide conservative confidence intervals.

Community-Level Data: In addition to sex, race, and age compositions, the following seven sociodemographic data were derived from the ACS 2012–2016 5-year estimates for each of the 537 Zip Code Tabulation Areas (ZCTA) of Massachusetts: % never married, % single householder, % with bachelor’s degree, % English spoken at home, % unemployed, % receiving food stamps, and per capita income.

Hospital Discharge Data (“Case Mix”): MA hospital discharge data due to asthma, diabetes (with or without complications), and hypertension (including essential and secondary) for those aged 18 and older were obtained from Massachusetts’ case mix database. Because hospitalizations due to a particular chronic illness are far rarer than prevalent within the population, we developed a calibration model for the discharge data and blended the resulting estimates with our SAE estimators.

Behavioral Risk Factor Surveillance System Data: Our gold standard for validating our model estimates was the CDC’s Behavioral Risk Factor Surveillance System (BRFSS) MA statewide and 500 Cities estimates. For many years, the BRFSS has been a mainstay of public health surveillance for chronic diseases [16]. This telephone survey generates national and state-specific estimates of the prevalence of the major chronic diseases and risk factors that state and local public health departments rely upon to monitor health, plan interventions, and monitor their impact. Recent innovations in BRFSS methods in response to evolving surveillance needs include adding mobile telephone numbers to the sample, imputing measures for the 500 largest cities, incorporating area-level poverty as a predictor, and fielding follow-up surveys to gather clinical care information for conditions such as asthma, diabetes, hypertension, obesity, and smoking. Despite this, BRFSS is disadvantaged by the limited number of questions asked, the self-reported nature of the data, the lack of clinical data, declining response rates [17], and differential nonresponse over sex, age, race/ethnicity, income, and rurality [18].

### MRP estimation procedure

We present comparisons of 2016 BRFSS statewide estimates from five candidate models:

- M_0_: Crude estimates with no adjustments
- M_1_: Adjustment for sex, race, age
- M_2_: M_1_ + American Community Survey
- ${\mathrm{M}}_1^{\mathrm{C}}$: M_1_ + calibrated Case Mix data
- ${\mathrm{M}}_2^{\mathrm{C}}$: M_2_ + calibrated Case Mix data

To test the influence of separate practice groups’ influence on the estimators, we fit M_0_, M_1_, M_2_ individually for each of the three practice groups and in aggregate; the aggregate model pooled the three separate fits by weighting the estimates by relative coverage by provider per ZCTA. This relative coverage was computed as the coverage of each provider divided by the coverage of all three providers and therefore the three relative coverages sum up to 100% (see Step 3 below for more details). We included this relative coverage to account for the large differences in disease prevalence rates amongst different providers; therefore, provider affiliation was used as a proxy for additional behavioral and sociodemographic characteristics associated with disease risk. We note that for models M_1_ and M_2_, we also weighted each race, age, and sex strata by the corresponding census weight in a procedure known as poststratification [9]. We did not fit ${\mathrm{M}}_1^{\mathrm{C}}$ nor ${\mathrm{M}}_2^{\mathrm{C}}$ for each provider because the calibration procedure, as described shortly, needs sufficient coverage per provider over the available ZCTAs which was not the situation. The estimation steps are described briefly below, and more mathematical details provided in Additional File 2. All analyses were performed using R version 3.6.1.

- Step 1 (Crude estimation of ZCTA-level disease prevalence per practice group): Direct prevalence estimates of the disease outcomes were computed based only off the available patients within each practice group in each ZCTA; many ZCTAs were inestimable due to lack of coverage from any of the three providers. This step forms M_0_ for Atrius, CHA, and MLCHC separately.
- Step 2 (Adjusted estimation of ZCTA-level disease prevalence per practice group): A generalized linear mixed model (GLMM) was used to estimate ZCTA-level prevalence for each outcome within each practice group as a function of predictors. Model M_1_ included sex, race, and age while M_2_ included the full set of predictors listed under Community Level Data.
- Step 3 (Pooling of crude and adjusted estimates from practice groups): Models M_0_, M_1_, M_2_ were then averaged over providers according to a relative coverage weighting, the weights were computed in each ZCTA as the number of patients subscribed to each provider divided by the total number subscribed to all three providers in that particular ZCTA. Stratum-specific weights were computed in a similar fashion. If data were not available for a stratum within a ZCTA, we rolled-up and replaced with the relative coverage for the overall ZCTA. If no data existed for the ZCTA, we rolled-up once again and used the overall relative coverage of the three data sources. This step forms M_0_, M_1_, M_2_ over all three providers.
- Step 4 (Incorporation of case mix data with pooled): Hospitalization data was available for three of the five disease outcomes (asthma, diabetes, hypertension). These data contain rates of hospitalization due to a specified complication as measured by number of hospitalizations with that specified complication divided by the population of each ZCTA over the years 2011–2015. Hospitalizations do not equate to general prevalences; we therefore calibrated these crude hospitalization proportions such that the overall proportions for MA matched the overall predictions from M_1_ and M_2_ and the variation of proportions over ZCTAs matched the variation from M_1_ and M_2_ predictions. This formed ${\mathrm{M}}_1^{\mathrm{C}}$ and ${\mathrm{M}}_2^{\mathrm{C}}$, our calibrated case mix estimates. For the other two outcomes without available case mix (obesity and smoking), their model values are deferred to M_1_ and M_2_.
- Step 5 (Roll-up from ZCTAs to municipalities and statewide estimates): The 537 ZCTAs of MA were aggregated to the 351 municipalities of MA and to the entirety of MA.

### Ranking municipalities

Ranking was determined by the following two-step procedure. First, the interquartile range (IQR) outlier detection test retains municipalities if their prevalence is greater than Q_3_ + 1.5(Q_3_ − Q_1_), where Q_1_ is the first quartile (25 percentile) and Q_3_ is the third quartile (75 percentile). Next, this shortlist is ordered based off the lower bound in a 95% confidence interval for the true proportion in that municipality; this system is known as Wilson score ranking and accounts for high prevalences which may be due to small sample sizes.

## Results

**Fig. 1:**
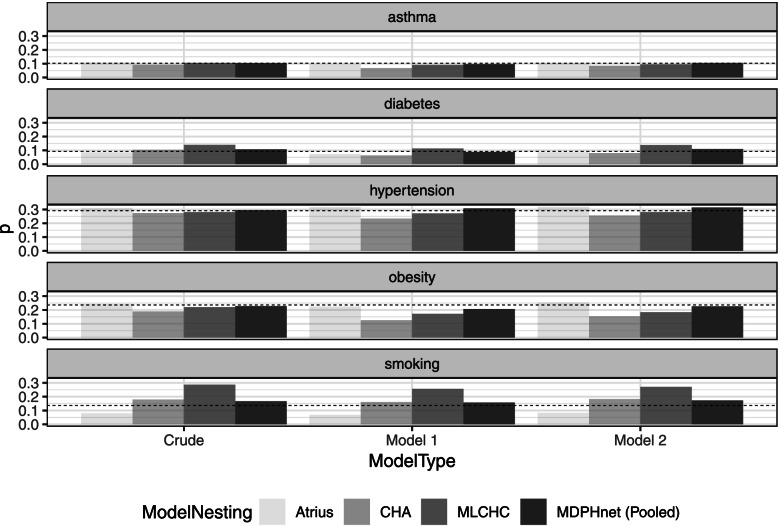
Comparison of increasingly refined model predictions from MDPHnet vs BRFSS estimates: Massachusetts (2016) statewide aggregate. Model 1 is the sex-race-age poststratification with coverage weighting. Model 2 is the fully loaded model with coverage weighting

Statewide estimates of the prevalence of asthma, diabetes, hypertension, obesity, and smoking from models M_0_, M_1_, M_2_ vs. BRFSS are presented in Fig. 1. ${\mathrm{M}}_1^{\mathrm{C}}$ and ${\mathrm{M}}_2^{\mathrm{C}}$ are not included in Fig. 1 because they were calibrated to have matching statewide estimates as M_1_ and M_2_. The pooled estimates from M_0_, M_1_, M_2_ closely matched BRFSS statewide estimates. However, estimates differed widely between practices, indicating substantial between-practice differences that did not resolve even after accounting for sociodemographic characteristics. Indeed, for each model M_0_, M_1_, and M_2_, the ranges (largest provider prediction minus smallest) were as follows: asthma (1.61, 2.78, 1.99%), diabetes (5.40, 5.00, 5.84%), hypertension (3.49, 8.39, 6.25%), obesity (5.27, 9.86, 10.1%), and smoking (20.6, 18.8, 18.8%), indicating that obesity and smoking estimates are very provider-dependent. In addition to aggregate results, we benchmarked MDPHnet predictions against the 2016 BRFSS / CDC 500 Cities estimates for cities found within MA, namely Boston, Brockton, Cambridge, Fall River, Lawrence, Lowell, Lynn, New Bedford, Newton, Quincy, Somerville, Springfield, and Worcester.

Tables 1 and 2 provide mean absolute error (MAE) and correlation coefficient (R) metrics to assess concordance between MDPHnet-based estimates and BRFSS. MAE and R represent two different qualities: MAE computes the averaged positive errors and therefore larger detractions from zero indicate greater differences in sampling methodology, target population definitions, model assumptions, etc. between MDPHnet estimates and BRFSS estimates; R characterizes relative agreement between MDPHnet estimates and BRFSS estimates, and therefore a higher R indicates MDPHnet would provide a more similar list of “hotspot” municipalities to that provided by BRFSS based off rankings despite differences in actual disease prevalences.

**Table 1:** Mean absolute error (percentage points) for various model fits vs. 2016 BRFSS 500 Cities estimates

		***M***_**0**_	***M***_**1**_	***M***_**2**_	${\boldsymbol{M}}_{\mathbf{1}}^{\mathbf{C}}$	${\boldsymbol{M}}_{\mathbf{2}}^{\mathbf{C}}$
**Asthma**	**Atrius**	1.97	2.05	0.82	–	–
	**CHA**	2.70	4.82	2.29	–	–
	**MLCHC**	3.30	2.51	1.91	–	–
	**MDPHnet**	2.24	1.83	1.02	1.35	0.61
**Diabetes**	**Atrius**	2.45	3.46	1.38	–	–
	**CHA**	2.86	4.81	2.88	–	–
	**MLCHC**	4.35	2.33	5.31	–	–
	**MDPHnet**	3.13	1.84	3.48	1.30	3.68
**Hypertension**	**Atrius**	4.82	2.18	2.46	–	–
	**CHA**	5.62	7.94	4.02	–	–
	**MLCHC**	3.53	3.25	2.22	–	–
	**MDPHnet**	2.60	2.33	1.48	1.33	2.23
**Obesity**	**Atrius**	5.29	5.13	2.99	–	–
	**CHA**	8.68	15.2	9.06	–	–
	**MLCHC**	6.55	9.64	6.95	–	–
	**MDPHnet**	4.92	6.92	4.07	6.92	4.07
**Smoking**	**Atrius**	9.15	12.47	8.94	–	–
	**CHA**	3.08	5.80	3.54	–	–
	**MLCHC**	8.92	6.60	8.53	–	–
	**MDPHnet**	5.33	2.84	2.99	2.84	2.99

**Table 2:** Correlation coefficients (out of 100) for various model fits vs. 2016 BRFSS 500 Cities estimates

		***M***_**0**_	***M***_**1**_	***M***_**2**_	${\boldsymbol{M}}_{\mathbf{1}}^{\mathbf{C}}$	${\boldsymbol{M}}_{\mathbf{2}}^{\mathbf{C}}$
**Asthma**	**Atrius**	24.1	−7.4	68.8	–	–
	**CHA**	23.9	− 36.5	45	–	–
	**MLCHC**	57.3	77.7	90.9	–	–
	**MDPHnet**	40.8	55.3	88.9	92.6	96.0
**Diabetes**	**Atrius**	51.8	−0.6	82.6	–	–
	**CHA**	0.4	−68.6	48.5	–	–
	**MLCHC**	45.1	7.5	90.8	–	–
	**MDPHnet**	67.1	77.1	96.7	83.3	91.5
**Hypertension**	**Atrius**	54.6	83.7	85.5	–	–
	**CHA**	−0.4	−20.6	59.9	–	–
	**MLCHC**	86.6	94.2	96.7	–	–
	**MDPHnet**	76.5	85.9	92.9	96.7	93.7
**Obesity**	**Atrius**	77.6	70.6	88.3	–	–
	**CHA**	51	−37.0	52.5	–	–
	**MLCHC**	56.4	83.3	86.6	–	–
	**MDPHnet**	57.5	86.7	81.9	83.3	86.6
**Smoking**	**Atrius**	81	30	73.6	–	–
	**CHA**	73.9	14.4	70.2	–	–
	**MLCHC**	52.5	68.6	86.7	–	–
	**MDPHnet**	82.5	85.8	93.9	85.8	93.9

**Fig. 2:**
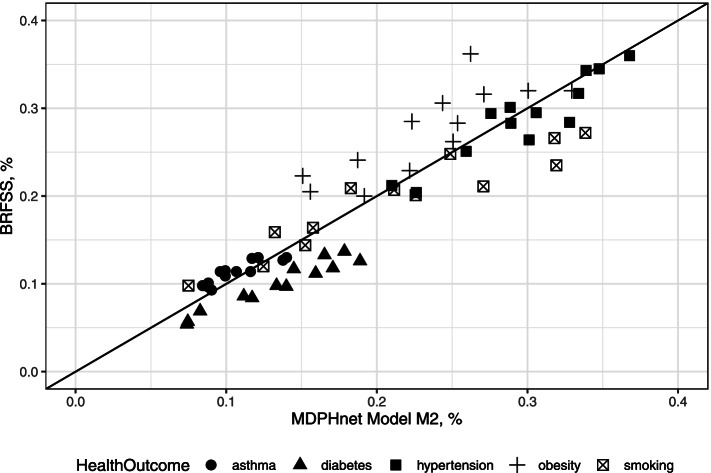
Comparison of MDPHnet M_2_ vs BRFSS: Massachusetts (2016) small-area estimates within the 13 overlapping municipalities from the 2016 BRFSS 500 Cities. Each marker indicates a single location and condition. The diagonal line marks where perfect agreement between MDPHnet and BRFSS would lie

**Fig. 3:**
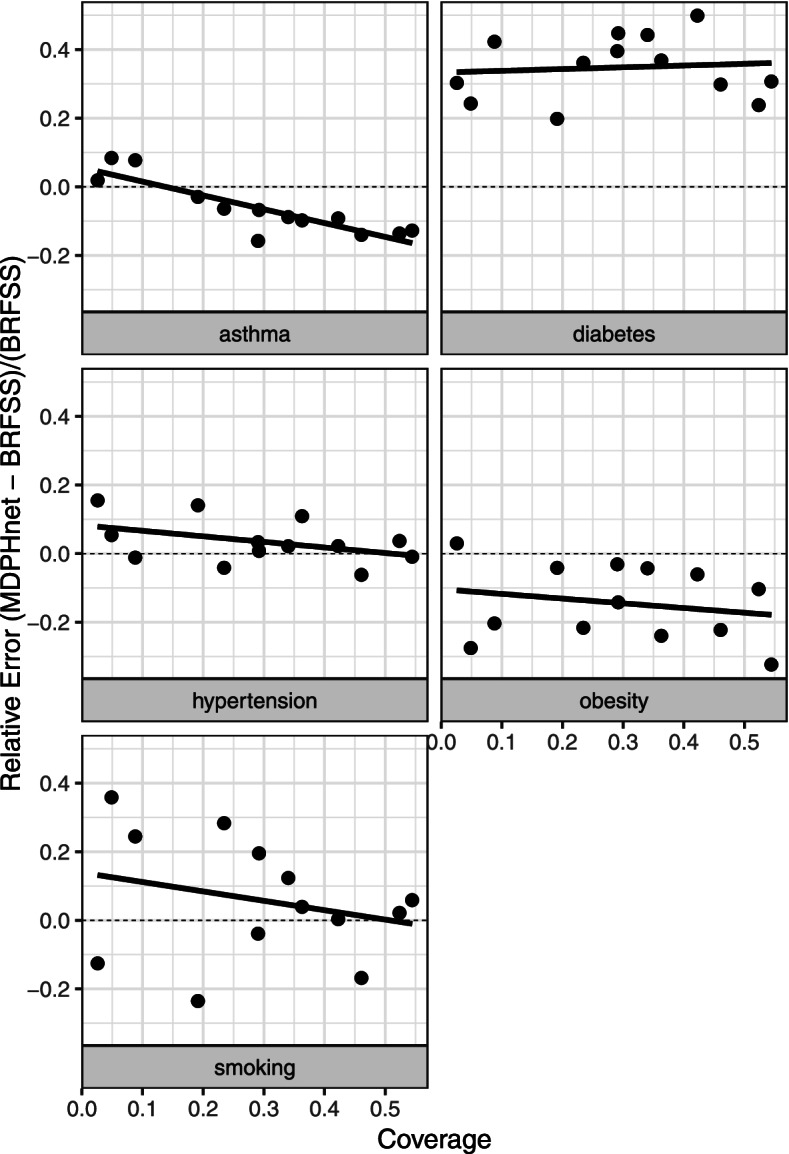
Relative error of MDPHnet M_2_ from BRFSS estimates vs MDPHnet coverage within the 13 overlapping municipalities from the 2016 BRFSS 500 cities

For the vast majority of target cities, M_2_ provided the closest concordance with BRFSS estimates using both MAE and R; case mix adjustment did not add much. For M_2_, we see that these two sets of estimates were especially close for asthma, diabetes, hypertension, and smoking but less so for obesity. The MAEs between M_2_ and BRFSS were asthma (1.02%), diabetes (3.48%), hypertension (1.48%), obesity (4.07%), and smoking (2.99%). The R^2^ between model (3) and BRFSS were asthma (88.9%), diabetes (96.7%), hypertension (92.9%), obesity (81.9%), smoking (93.9%), which all represent strong agreement in the ranking of municipality outcome prevalences. Furthermore, Table 2 provides additional evidence of strong associations between choice of providers and health outcomes, with varying correlations for each provider-specific estimates vs BRFSS estimates. Figure 2 provides a graphical representation of the information provided in Tables 1 and 2. Within each disease category, MAE is the average of the distances from the diagonal line and R^2^ characterizes how colinear (and therefore, how concordant) the estimates are between M_2_ and BRFSS. We note that MDPHnet coverage of an area also heavily influences predictions; Fig. 3 indicates a downward difference in MDPHnet estimates vs BRFSS estimates as coverage increases.

In addition to small-area estimates for larger municipalities found within MA, we use M_2_ to estimate the prevalences of each of the 5 target conditions for the remaining 338 municipalities in Massachusetts and to pinpoint those with the highest prevalence rates (Additional file 3 for this list of predictions). Table 3 contains five subtables corresponding to each health outcome and lists the top five at-risk municipalities under the methods described in Ranking municipalities Section and based off predictions from M_2_. From this procedure, we identified 17 municipalities for asthma, 11 for diabetes, 1 for hypertension, 4 for obesity, and none for smoking.

**Table 3:** Rankings of highest at-risk municipalities for each disease outcome

Rank	Municipality	2012–2016 ACS Population	Prevalence	Confidence Interval
	(a)	Top 5 MA asthma-risk municipalities by Wilson lower bound		
1	Revere	42,125	0.170	(0.167, 0.174)
2	Orange	6061	0.167	(0.157, 0.176)
3	Malden	48,521	0.155	(0.151, 0.158)
4	Chelsea	27,849	0.149	(0.145, 0.154)
5	North Adams	12,390	0.145	(0.139, 0.151)
	(b)	Top 5 MA diabetes-risk municipalities by Wilson lower bound		
1	Brockton	67,992	0.189	(0.186, 0.192)
2	Everett	33,062	0.188	(0.183, 0.192)
3	Lawrence	55,244	0.178	(0.175, 0.182)
4	Chelsea	27,849	0.174	(0.170, 0.178)
5	New Bedford	71,200	0.171	(0.168, 0.174)
	(c)	Top 5 MA hypertension-risk municipalities by Wilson lower bound		
1	Lenox	4094	0.435	(0.420, 0.450)
	(d)	Top 5 MA obesity-risk municipalities by Wilson lower bound		
1	Lawrence	55,244	0.329	(0.326, 0.333)
2	Chelsea	27,849	0.324	(0.318, 0.329)
3	Randolph	26,684	0.304	(0.298, 0.309)
4	Brockton	67,992	0.301	(0.297, 0.304)
	(e)	Top 5 MA smoking-risk municipalities by Wilson lower bound		
NA	NA	NA	NA	NA

## Discussion

This paper demonstrates small-area estimation techniques are useful to hone EHR-based disease prevalence estimates for areas that are underrepresented in EHR data and can pinpoint communities with statistically higher disease rates to help public health agencies target interventions. In general, the inclusion of socioeconomic and community data fine-tunes estimates because it constitutes ecological factors which are associated with health outcomes. We found that including sociodemographic factors improved agreement with BRFSS estimates above and beyond adjusting for sex, race, age, and relative coverage and was far superior to a crude model. Intuitively, a model fit with more data from a target ZCTA should be given more weight in its prediction, which our relative coverage weighting exactly stipulates.

Compared to traditional health surveys, EHRs offer larger and more pertinent data thus potentially offering useful insights for real-time chronic disease monitoring and expected trends. The use of EHRs for public health surveillance has been limited due to concerns about the non-random coverage of patients. We demonstrate that EHR-based estimates drawn from multiple practice groups and adjusted using individual demographics, the relative contribution by practice, and community-level socioeconomic data can provide disease prevalence estimates that are very similar to small area estimates derived from traditional health surveys. This may allow for broader utilization of EHR data to facilitate timely and detailed public health surveillance for both large and small areas.

### Strengths and limitations

MDPHnet demonstrates the great potential for EHR-based chronic disease surveillance. EHR-based surveillance can monitor large populations with clinical information such as diagnosis and pharmacy data. The electronic nature of the data facilitates real-time statistical analysis. However, EHRs contain information only on those people receiving health care, who may have characteristics different from those not receiving health care. Because the proportion of a population receiving care may depend in part on health insurance coverage, EHR-based surveillance may perform worse in jurisdictions with lower insurance coverage. In addition, MDPHnet data suggest that the validity of small-area estimates may vary depending on the proportion of the local population covered by the EHR and the number of participating providers. Small-area estimates may be imprecise if the participating EHRs cover only a small portion of the population. Ultimately, this concern can be mitigated by adding more practice groups’ EHR data into existing surveillance systems and therefore increasing the proportion of the population under surveillance. Improved EHR coverage would be the first step in improving record keeping of healthcare-seeking behaviors, access, afforability, and insurance coverage. To the extent one can measure these metrics, incorporating them into SAE models would better inform predictions. Further work is necessary to determine the impact of these factors and to validate EHR-based estimates for racial/ethnic subgroups, as modeling based on just demographic characteristics alone is not enough.

Finally, EHR-based surveillance cannot replace traditional population-based surveillance surveys like BRFSS. Surveillance surveys collect data on health behaviors and self-perceived health status in the general population which are not typically available in EHRs (such as exercise, diet, and well-being) as well as data on people not in care. Nevertheless, while we treated BRFSS as the gold standard in this exercise, surveys continue to encounter selection bias and the concordance between BRFSS and MDPHnet SAE estimates does not necessarily exhibit the true chronic disease prevelances.

## Conclusions

Development of SAEs can be critical during an emerging public health event, especially one distributed over a wide geographic area. In MA and other jurisdictions, survey response rates are declining, and results from traditional surveillance systems are unreliable for smaller municipalities or ZCTAs because these areas do not have sufficient samples to produce robust estimates. While EHR-based surveillance is still in its infancy, replicating and extending the models developed in this paper offer prospects of detailed and timely public health surveillance for small and large jurisdictions across the country. Standardizing methods and algorithms and sharing technological solutions will help facilitate this work.

The methods in this work were adapted from statistical techniques that have been used for identifying individual- and community-level risk factors. Our approach is straightforward and cost-effective, can be easily translated into routine practice and implemented with existing data via readily available statistical software packages. Proper adaptation of these methods may expand the scope of existing national health surveillance and data collection systems and provide health information and specific recommendations that are directly relevant to local communities and governments.

## Supplementary Information

**Additional file 1.** MDPHnet Disease Identification Criteria. Conditions disease definitions for asthma, diabetes, hypertension, obesity, smoking.

**Additional file 2.** Details of the Estimation Procedure. Contains more statistical details regarding the estimation procedure discussed in MRP estimation procedure Section.

**Additional file 3. **Predictions from each model (*M*_0_, *M*_1_, *M*_2_) for each test condition (asthma, diabetes, hypertension, obesity, smoking) for each practice (Atrius, CHA, MLCHC, aggregate) in each of the 351 municipalities.

## Data Availability

MDPHnet and hospital discharge data are not publicly available but are available from the corresponding author upon reasonable request. BRFSS 2016 estimates are available from the Centers for Disease Control and Prevention [https://chronicdata.cdc.gov/500-Cities-Places/500-Cities-Local-Data-for-Better-Health-2018-relea/rja3-32tc]. 2012–2016 ACS data are available from the US Census Bureau [https://www.census.gov/acs/www/data/data-tables-and-tools/data-profiles/2016/].

## Abbreviations

ACS: American Community Survey
BRFSS: Behavioral Risk Factor Surveillance System
CDC: Centers for Disease Control and Prevention
CHA: Cambridge Health Alliance
EHR: Electronic health records
IQR: Interquartile range
MA: Massachusetts
MAE: Mean absolute error
MLCHC: Massachusetts League of Community Health Centers
SAE: Small-area estimation
ZCTA: ZIP code tabulation area
