# Supplementary material for: Small-area estimation for public health surveillance using electronic health record data: reducing the impact of underrepresentation
Source: BMC Public Health. 2022 Aug 9;22:1515. doi: 10.1186/s12889-022-13809-2 (PMC9364501; doi:10.1186/s12889-022-13809-2)
Supplement: Supplementary file 1 — Additional file 1. MDPHnet Disease Identification Criteria. Conditions disease definitions for asthma, diabetes, hypertension, obesity, smoking. [file 12889_2022_13809_MOESM1_ESM.docx]

MDPHnet Disease Identification Criteria

| *Condition and Definition* |
| --- |
| Diabetes (≥ 1 of the following): |
| - Hemoglobin A1C ≥ 6.5 |
| - Fasting glucose ≥ 126 |
| - Random glucose ≥ 200 on 2 or more occasions |
| - Prescription for insulin outside of pregnancy |
| - ICD-9 code 250.xx or ICD-10 code E10*, E11*, or E14* on 2 or more occasions |
| - Prescription for glyburide, gliclazide, glipizide, glimepiride, pioglitazone, rosiglitazone, repaglinide, nateglinide, meglitinide, sitagliptin, exenatide, alogliptin, linagliptin, saxagliptin, albiglutide, dulaglutide, liraglutide, canagliflozin, dapagliflozin, empagliflozin, or pramlintide |
| Asthma (≥ 1 of the following): |
| - ≥ 2 encounters with diagnosis code for asthma (ICD-9 493.xx or ICD-10 J45*–J46*) |
| - ≥ 2 prescriptions for albuterol, levalbuterol, pirbuterol, arformoterol, formoterol, indacaterol, salmeterol, beclomethasone, inhaled budesonide, inhaled ciclesonide, inhaled flunisolide, inhaled fluticasone, inhaled mometasone, montelukast, zafirlukast, zileuton, ipratropium, tiotropium, cromolyn INH, omalizumab, fluticasone + salmeterol, albuterol + ipratropium, mometasone + formoterol, or budesonide + formoterol |
| Smoking: |
| - Most recent smoking status of “current” as recorded in the EHR “tobacco” field (synonyms for current acceptable) |
| Hypertension (≥ 1 of the following): |
| - Systolic blood pressure ≥ 140 mm Hg or diastolic blood pressure ≥ 90 on ≥ 2 occasions within a 1-y period |
| - Diagnosis code for hypertension (ICD-9 401.xx or 405.xx or ICD-10 I10 or I15) and prescription for at least 1 of the following medications within 1 y of the hypertension diagnosis code: hydrochlorothiazide, indapamide, amlodipine, clevidipine, felodipine, isradipine, nicardipine, nifedipine, nisoldipine, diltiazem, verapamil, acebutolol, atenolol, betaxolol, bisoprolol, carvedilol, labetolol, metoprolol, nadolol, nebivolol, pindolol, propranolol, benazepril, captopril, enalapril, fosinopril, lisinopril, moexipril, perindopril, quinapril, ramipril, trandolapril, candesartan, eprosartan, irbesartan, losartan, olmesartan, telmisartan, valsartan, clonidine, doxazosin, guanfacine, or methyldopa |
| Obesity: |
| - Most recent BMI ≥ 30 kg/m2 |
| - If BMI not available, calculate using last available weight measured within the last y and last available height measured when aged ≥ 16 y |
