# Supplementary material for: Small-area estimation for public health surveillance using electronic health record data: reducing the impact of underrepresentation
Source: BMC Public Health. 2022 Aug 9;22:1515. doi: 10.1186/s12889-022-13809-2 (PMC9364501; doi:10.1186/s12889-022-13809-2)
Supplement: Supplementary file 2 — Additional file 2. Details of the Estimation Procedure. Contains more statistical details regarding the estimation procedure discussed in MRP estimation procedure Section. [file 12889_2022_13809_MOESM2_ESM.docx]

Details of the Estimation Procedure

Step 1 (*Crude estimation of ZCTA-level disease prevalence per practice group)*

Denote $Y=0,1$ be the dichotomous measure of the presence or absence of a disease outcome, and suppose that $Y_{pzi}$ is a Bernoulli variable denoting the disease outcome for individual $i$ within ZCTA $z$ and provider $p$. Because the data are de-identified, there is no way to tell which individuals are carried over between months and therefore data from different months are assumed to be different individuals. The crude estimates within ZCTA $z$ and healthcare provider $h$is computing as

$$\hat{p}_{hz}^{M_{0}}=\frac{1}{m_{hz}}\sum_{i=1}^{m_{hz}} Y_{hzi}$$

i.e. the direct averages of individuals within each $h$ and $p$, where $m_{hz}$ is the number of individuals in ZCTA $z$ within provider $h$.

Step 2 *(Adjusted estimation of ZCTA-level disease prevalence per practice group)*

We used a random-effects logistic regression model to estimate for each of the 537 MA ZCTAs the probability that a person in the ZCTA has a disease outcome during each month as a function of personal level variables and ecological variables specific to that ZCTA. The model is as follows:

$$\mathrm{logit}\left( p_{hzi}^{M} \right)=\delta_{h}+\alpha_{h}t+\beta_{h}^{T}X_{hzi}+\gamma_{h}^{T}Z_{hz}+U_{hz}$$

where $M=M_{1},M_{2}$ specifying community-level covariates from ACS is not or is included, resptively, $p_{hzi}^{M}=P\left( Y_{hzi}=1 | X_{hzi},Z_{hzi} \right)$, $t$ is the month of 2016, $X_{hzi}$ is a vector of $d_{1}$ individual level covariates, $Z_{hz}$ is a vector of $d_{2}$ community level covariates, and $\alpha_{h},\beta_{h},\gamma_{h}$ are their respective coefficients with $\delta_{h}$ being the intercept term. In our specific model, we have $d_{1}=3$ individual level covariates and $d_{2}=7$ community level covariates, although some of them are categorical and therefore expand to multiple factors each needing a separate coefficient. The random effect $U_{hz}$ was assumed to be independent and normally distributed with mean zero and variance $\sigma_{h}^{2}$. After fitting this model, we are equipped with parameter estimates $\hat{\delta}_{h},\hat{\alpha}_{h},\hat{\beta}_{h},\hat{\gamma}_{h}$. The adjusted estimates within ZCTA $z$ and healthcare provider $h$ then provided by the following post-stratification estimate:

$$\hat{p}_{hz}^{M}=\sum_{s=1}^{120} w_{zs}\mathrm{logit}^{-1}\left[ \hat{\delta}_{h}+\hat{\alpha}_{h}\cdot6+\hat{\beta}_{h}^{T}X_{zs}+\hat{\gamma}_{h}^{T}\bar{Z}_{z} \right]$$

where $w_{zs}$ is the weight for strata $s$ of the 120 combinations of sex (male/female), race (white, black, Asian, Hispanic, other), and age (0-4, 5-9, 10-14, 15-19, 20-24, 25-29, 30-39, 40-49, 50-59, 60-69, 70-79, ≥ 80) determined from ACS within ZCTA $z$, and $X_{zs}$ is its corresponding value of sex, race, and age. As mentioned in the main article, because other characteristics besides sex, race, and age are not available in MDPHnet, we set $\bar{Z}_{z}$ to be the average of the community level characteristics.

Step 3 (*Pooling of crude and adjusted estimates from practice groups*)

We blend each provider-specific prevalence estimate based on relative coverage:

$$\hat{p}_{z}^{M}=w_{1z}\hat{p}_{1z}^{M}+w_{2z}\hat{p}_{2z}^{M}+w_{3z}\hat{p}_{3z}^{M}$$

where $w_{hz}$ is the relative coverage for provider $h$ within ZCTA $z$ which satisfy $w_{1z}+w_{2z}+w_{3z}=1$, and $M=M_{0},M_{1},M_{2}$. Note that these weights can be quite sparse; if no data exists for a particular strata within ZCTA $i$, we roll-up and replace with the relative coverage for the overall ZCTA $i$. If no data exists for the ZCTA, we roll-up once again and use the overall relative coverage of the three data sources.

Step 4 *(Incorporation of case mix data with pooled)*

For outcomes in which case mix data is available (which are asthma, diabetes, and hypertension), let $\hat{p}_{z}^{cm}$ be estimated prevalences of disease outcomes from case mix data. Because these proportions are recorded hospitalizations as a result of complications due to the disease, it will underestimate the true prevalences in a population. However, if hospitalizations are believed to be proportional to population size, we surmise some monotone increasing transformation of $\hat{p}_{i}^{cm}$ to produce the true prevalences:

$$\hat{p}_{z}^{ccm}=f\left( \hat{p}_{z}^{cm} \right)$$

where $ccm$ denotes calibrated case mix. The specific functional form we take for $f\left( \cdot\right)$ is

$$f\left( p \right)=\mathrm{logit}^{-1}\left[ a\cdot\mathrm{logit}\left( p \right)+b \right]$$

That is, crude case mix proportions are transformed to the logit scale, a linear transformation is performed, and then this result is transformed back onto the probability scale. The parameters $a,b$ are calibrated so that

$$\bar{p}^{ccm}≝\frac{1}{N}\sum_{z=1}^{537} N_{z}\hat{p}_{z}^{ccm}=\frac{1}{N}\sum_{z=1}^{537} N_{z}\hat{p}_{z}^{M}≝\bar{p}^{M}$$

and

$$\frac{1}{N}\sum_{z=1}^{537} N_{z}\left( \hat{p}_{z}^{ccm}-\bar{p}^{ccm} \right)^{2}=\frac{1}{N}\sum_{z=1}^{537} N_{z}\left( \hat{p}_{z}^{M}-\bar{p}^{M} \right)^{2}$$

where $N_{z}$ is the ACS population for ZCTA $z$ and $N=\sum_{z=1}^{537} N_{z}$. That is, the overall prevalence and the between-ZCTA variances are matched between the calibrated case mix and model-assisted estimates. The aggregated estimates for each ZCTA will be an equal parts blend of the model-assisted estimates and calibrated case mix:

$$\hat{p}_{z}=\frac{1}{2}\bar{p}_{z}^{M}+\frac{1}{2}\hat{p}_{z}^{ccm}$$

if case mix data were available for the disease outcome, otherwise $\hat{p}_{i}=\bar{p}_{z}^{M}$.

Step 5 *(Roll-up from ZCTAs to municipalities and statewide estimates)*

Let $\hat{q}_{l}$ be estimated disease prevalences for municipality $l$ and let $z\in l$ denote all ZCTAs which fall within municipality $l$. Then the municipality-level prevelances are computed as

$$\hat{q}_{m}=\frac{\sum_{z\in m} N_{z}\hat{p}_{z}}{\sum_{z\in m} N_{z}}$$

and statewide prevalence computed as

$$\hat{q}=\frac{\sum_{z=1}^{537} N_{z}\hat{p}_{z}}{\sum_{z=1}^{537} N_{z}}$$
